# Diversity of Sulfated Polysaccharides From Cell Walls of Coenocytic Green Algae and Their Structural Relationships in View of Green Algal Evolution

**DOI:** 10.3389/fpls.2020.554585

**Published:** 2020-09-29

**Authors:** Marina Ciancia, Paula Virginia Fernández, Frederik Leliaert

**Affiliations:** ^1^Universidad de Buenos Aires, Facultad de Agronomía, Departamento de Biología Aplicada y Alimentos, Cátedra de Química de Biomoléculas, CIHIDECAR-CONICET, UBA, Buenos Aires, Argentina; ^2^Meise Botanic Garden, Meise, Belgium; ^3^Ghent University, Faculty of Sciences, Biology Department, Ghent, Belgium

**Keywords:** coenocytic green algae, sulfated galactan, sulfated arabinan, phylogeny, Ulvophyceae, cell wall

## Abstract

Seaweeds biosynthesize sulfated polysaccharides as key components of their cell walls. These polysaccharides are potentially interesting as biologically active compounds. Green macroalgae of the class Ulvophyceae comprise sulfated polysaccharides with great structural differences regarding the monosaccharide constituents, linearity of their backbones, and presence of other acidic substituents in their structure, including uronic acid residues and pyruvic acid. These structures have been thoroughly studied in the Ulvales and Ulotrichales, but only more recently have they been investigated with some detail in ulvophytes with giant multinucleate (coenocytic) cells, including the siphonous Bryopsidales and Dasycladales, and the siphonocladous Cladophorales. An early classification of these structurally heterogeneous polysaccharides was based on the presence of uronic acid residues in these molecules. In agreement with this classification based on chemical structures, sulfated polysaccharides of the orders Bryopsidales and Cladophorales fall in the same group, in which this acidic component is absent, or only present in very low quantities. The cell walls of Dasycladales have been less studied, and it remains unclear if they comprise sulfated polysaccharides of both types. Although in the Bryopsidales and Cladophorales the most important sulfated polysaccharides are arabinans and galactans (or arabinogalactans), their major structures are very different. The Bryopsidales produce sulfated pyruvylated 3-linked β-d-galactans, in most cases, with ramifications on C6. For some species, linear sulfated pyranosic β-l-arabinans have been described. In the Cladophorales, also sulfated pyranosic β-l-arabinans have been found, but 4-linked and highly substituted with side chains. These differences are consistent with recent molecular phylogenetic analyses, which indicate that the Bryopsidales and Cladophorales are distantly related. In addition, some of the Bryopsidales also biosynthesize other sulfated polysaccharides, i.e., sulfated mannans and sulfated rhamnans. The presence of sulfate groups as a distinctive characteristic of these biopolymers has been related to their adaptation to the marine environment. However, it has been shown that some freshwater algae from the Cladophorales also produce sulfated polysaccharides. In this review, structures of sulfated polysaccharides from bryopsidalean, dasycladalean, and cladophoralean green algae studied until now are described and analyzed based on current phylogenetic understanding, with the aim of unveiling the important knowledge gaps that still exist.

## Introduction

Sulfated polysaccharides encompass a diverse group of anionic polymers, occurring in many different groups of organisms, from macroalgae to mammals, but they are not found in land plants. For marine organisms, as seaweeds, marine invertebrates and sea grasses, sulfated polysaccharides are supposed to be a physiological adaptation to the high ionic strength of the marine environment (Kloareg and Quatrano, 1988; Aquino et al., 2005; Pomin and Mourao, 2008). In addition, they are believed to have important support and protective functions, for example through moisture retention capacity that enhances desiccation resistance (Kloareg and Quatrano, 1988; Arata et al., 2017a). They may also be involved in cell interaction and adhesion, and form a protective barrier against pathogens (Vavilala and D´Souza, 2015; Udayan et al., 2017). Seaweeds, including red (Rhodophyta), green (Chlorophyta), and brown (Phaeophyceae) marine macroalgae, biosynthesize sulfated polysaccharides as a key component of their cell walls (Cosenza et al., 2017). The amounts and structure of these cell wall components vary greatly. Well-known sulfated polysaccharides in seaweeds include galactans (agarans and carrageenans) from red algae, ulvans from green algae, and fucans and fucoidans from brown algae (Usov, 2011; Pangestuti and Kurnianto, 2017; Deniaud-Bouët et al., 2017). Algal sulfated polysaccharides are often complex and they are biosynthesized as heterogeneous mixtures (Pomin and Mourao, 2008), having composition and structure modulated by phylogenetic and environmental factors. From these mixtures, usually extracted from the alga by aqueous solvents, polysaccharide fractions, homogeneous with respect their structure and molecular weight, are difficult to obtain.

**Figure 1:**
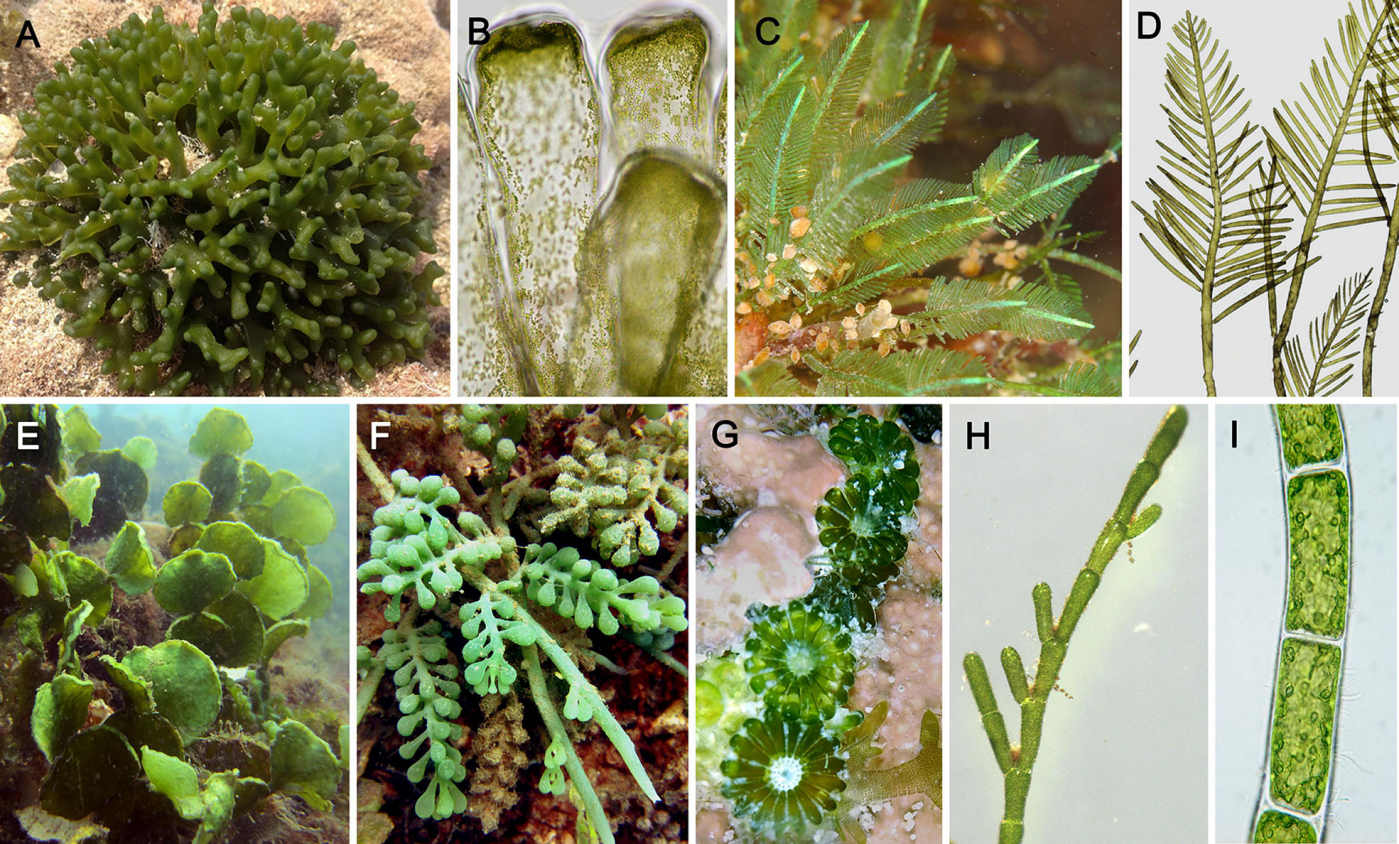
Morphological diversity among coenocytic green algae of the class Ulvophyceae. **(A)** *Codium* (Bryopsidales), forming spongy thalli composed of intertwined siphons and an outer layer of vesicles called utricles. **(B)** *Codium*, detail of utricles. **(C)** *Bryopsis* (Bryopsidales) composed of feather-like siphons. **(D)** *Bryopsis*, details. **(E)** *Halimeda* (Bryopsidales) forming complex thalli consisting of flattened calcified segments joint together by non-calcareous joints; segments composed of intertwined siphons and an outer layer of vesicles. **(F)** *Caulerpa* (Bryopsidales) forming large thalli composed of stolons anchored to the substrate by rhizoids, and upright photosynthetic fronds. **(G)** *Parvocaulis* (Dasycladales), forming thalli similar to *Acetabularia*, composed of a siphonous stalk and flattened terminal cap. **(H)** *Cladophora* (Cladophorales), consisting of branched filaments of multinucleate cells. **(I)** *Chaetomorpha* (Cladophorales), consisting of unbranched filaments. [Photo credits: **(A)** *Codium intricatum*, by Edward Steven, licensed under CC BY-SA 2.0, https://www.flickr.com/photos/138282132@N03/38293915915. **(B)** *Codium vermilara*, by Ignacio Barbara, licensed under CC BY-NC-SA 4.0, http://www.marbef.org/modules/photogallery/index.php?album=239&pic=15045. **(D–F, H, I)** Photo by F. Leliaert].

The Ulvophyceae include a wide range of marine macroalgae (green seaweeds), but several members also occur in freshwater or moist subaerial habitats. Species in the class display a wide variety of forms, ranging from microscopic unicellular algae to macroscopic multicellular algae, and giant-celled organisms with unique cellular and physiological characteristics (Mine et al., 2008). The origin and early diversification of Ulvophyceae likely took place in the late Tonian and Cryogenian periods, followed by a diversification of the main macroscopic clades in the Paleozoic (Del Cortona et al., 2020). Giant-celled green algae can be categorized into two cytomorphological types, the siphonous and siphonocladous types (both are also referred to as coenocytic). The siphonous type is characterized by macroscopic algae consisting of a single giant tubular cell generally containing thousands to millions of nuclei. It is present in the orders Bryopsidales and Dasycladales. Their cytoplasm exhibits vigorous streaming, enabling transportation of RNA transcripts across the plant. The siphonocladous type, that characterizes the Cladophorales, has multicellular bodies composed of multinucleate cells with nuclei organized in regularly spaced cytoplasmic domains (**Figure 1**).

**Figure 2:**
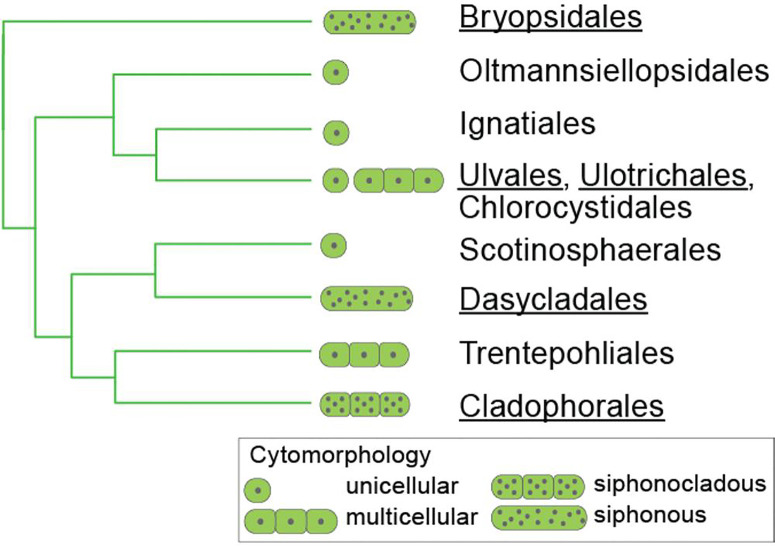
Relationships between the orders of Ulvophyceae, based on Del Cortona et al., 2020 and Škaloud et al., 2018. Those orders for which information on sulfated polysaccharides is available are underlined.

A solid phylogeny of the Ulvophyceae is important to understand the evolution traits, such as thallus morphology and cell wall structure and components. The relationships among the main clades of ulvophytes have long been uncertain, and even monophyly of the class has been under debate (reviewed by Del Cortona and Leliaert, 2018). A recent genome-scale phylogeny has largely resolved the relationships among the main clades of Ulvophyceae and indicated that macroscopic growth, as well as siphonous organization originated several times independently from unicellular ancestors (Del Cortona et al., 2020). **Figure 2** shows our current understanding of phylogenetic relationships between the different orders within the Ulvophyceae.

Species in the Ulvophyceae biosynthesize sulfated polysaccharides with important structural differences regarding the monosaccharide constituents, linearity of their backbones, presence of methoxyl groups, uronic acid residues, as well as acidic substituents of neutral monosaccharide units, including pyruvic acid, which contribute with sulfate groups to the negative charge of these polymers (Percival, 1979; Lahaye and Robic, 2007; Fernández et al., 2014; Stiger-Pouvreau et al., 2016; Kidgell et al., 2019).

An early classification of this structurally heterogeneous polysaccharide group, that is still very useful and generally used today, was based on the presence of uronic acid residues in their structures (Percival, 1979; Fernández et al., 2014; Stiger-Pouvreau et al., 2016).

Sulfated polysaccharides from the Ulvales and Ulotrichales have been thoroughly studied; they belong to the group comprising significant quantities of uronic acids. There are some major differences between the structures of those from both orders. Ulvans, the sulfated polysaccharides from the Ulvales, comprise major amounts of rhamnose and uronic acids disaccharidic repeating units. Many different oligosaccharides were obtained by hydrolysis of ulvans, a major structure found was 4-*O*-β-d-glucuronosyl-l-rhamnose. Also important quantities of other sugars were detected, as xylose, glucose, and iduronic acid (Lahaye and Robic, 2007). Sulfated polysaccharides from the Ulotrichales, as those from some species of the genus *Monostroma* and *Gayralia oxysperma* are also constituted by major amounts of rhamnose and glucuronic acid, but they have a rhamnose backbone with uronic acids as side chains (Cassolato et al., 2008; Liu et al., 2018).

Only more recently those from ulvophytes with coenocytic cells are beginning to be understood. In agreement with their chemical structures, sulfated polysaccharides of the orders Bryopsidales and Cladophorales fall in the same group, in which this acidic component is almost or completely absent in their matrix polysaccharides. Taking into account the phylogenetic affinity of Cladophorales and Dasycladales (Del Cortona et al., 2020), sulfated polysaccharides from the Dasycladales would be expected to biosynthesize polysaccharides belonging to the same group. However, cell wall components of Dasycladales remain poorly investigated. The study of Percival & Smestad (1972) suggests that dasycladalean species biosynthesize sulfated polysaccharide of both groups, or completely different polysaccharides with structural characteristics from both of them, although the results remain ambiguous (see later).

In the siphonous and siphonocladous green algae, sulfated polysaccharides are produced in very low quantities (ca. 1–20%), comparing with those from other algal groups, as red seaweed galactans (ca. 50%) (Venkata Rao and Sri Ramana, 1991; Falshaw et al., 2001; Estevez et al., 2004; Ciancia et al., 2007; Arata et al., 2015; Arata et al., 2016; Pérez Recalde et al., 2016).

The Bryopsidales are almost entirely restricted to marine habitats, except for a single genus, *Dichotomosiphon*, which also occurs in freshwater habitats. Some of the Bryopsidales have (1→4)-β-d-mannans as major fibrillar cell wall component, instead of cellulose. This important characteristic is shared with the Dasycladales, based on a study on *Acetabularia acetabulum* (Dunn et al., 2007). In particular, mannans were found to be the major fibrillar polysaccharides in *Codium* species, while major quantities of 3-linked β-d-xylans, plus small amounts of cellulose were found in *Bryopsis* and *Dichotomosiphon* (Maeda et al., 1966; Percival and McDowell, 1981; Fernández et al., 2014). It has been shown that the major fibrillar polysaccharides can vary in the different life stages of a single species. For example, sporophyte macrothalli of the genus *Derbesia* have fibrillar mannans as major components, while gametophyte microthalli biosynthesize xylans. Gametophyte macrothalli from the genus *Bryopsis* biosynthesize important quantities of xylans, while sporophyte microthalli produce mannans (Huizing and Rietema, 1975; Wutz and Zetsche, 1976; Huizing et al., 1979).

The Cladophorales are found in diverse habitats, ranging from marine habitats (occurring in the intertidal down to depths of more than 100 meters) to a wide range of freshwater habitats (Leliaert et al., 2012; Škaloud et al., 2018). Their cell walls are composed of cellulose I as major fibrillar polymer, with parallel microfibrils in numerous lamellae, of high crystallinity, lacking amorphous regions characteristic of plant cellulose (Zulkifly et al., 2013). In the cell walls of *Pithophora roettleri*, chitin was present in small amounts (>10%), in addition to cellulose (Pearlmutter and Lembi, 1980).

Although an important number of studies have been published regarding structural features of sulfated polysaccharides from seaweeds in the orders Bryopsidales, Cladophorales, and Dasycladales, information is much scarcer than that of galactans in cell walls of red seaweeds or fucoidans from brown seaweeds. Many clades within the three green seaweed orders have not been studied with respect to their water soluble cell wall polysaccharides, and in addition many other marine, freshwater, and terrestrial orders of the Ulvophyceae (e.g., Trentepohliales, Ignatiales, Scotinosphaerales, Chlorocystidales, and Oltmannsiellopsidales) have not been investigated at all. Hence, our knowledge of ulvophycean cell wall polysaccharides is scattered and incomplete.

In this paper, we review the current knowledge on structural diversity of sulfated polysaccharides from the three main orders of coenocytic green seaweeds, Bryopsidales, Cladophorales, and Dasycladales, and place these data in an evolutionary context based on current understanding of phylogenetic relationships. With this review, we aim to systematize the dispersed studies on these products, which are potentially interesting as biologically active compounds, considering current views on the phylogenetic relationships among the algae that produce them.

## Sulfated polysaccharides from coenocytic green algae

### Bryopsidales

**Figure 3:**
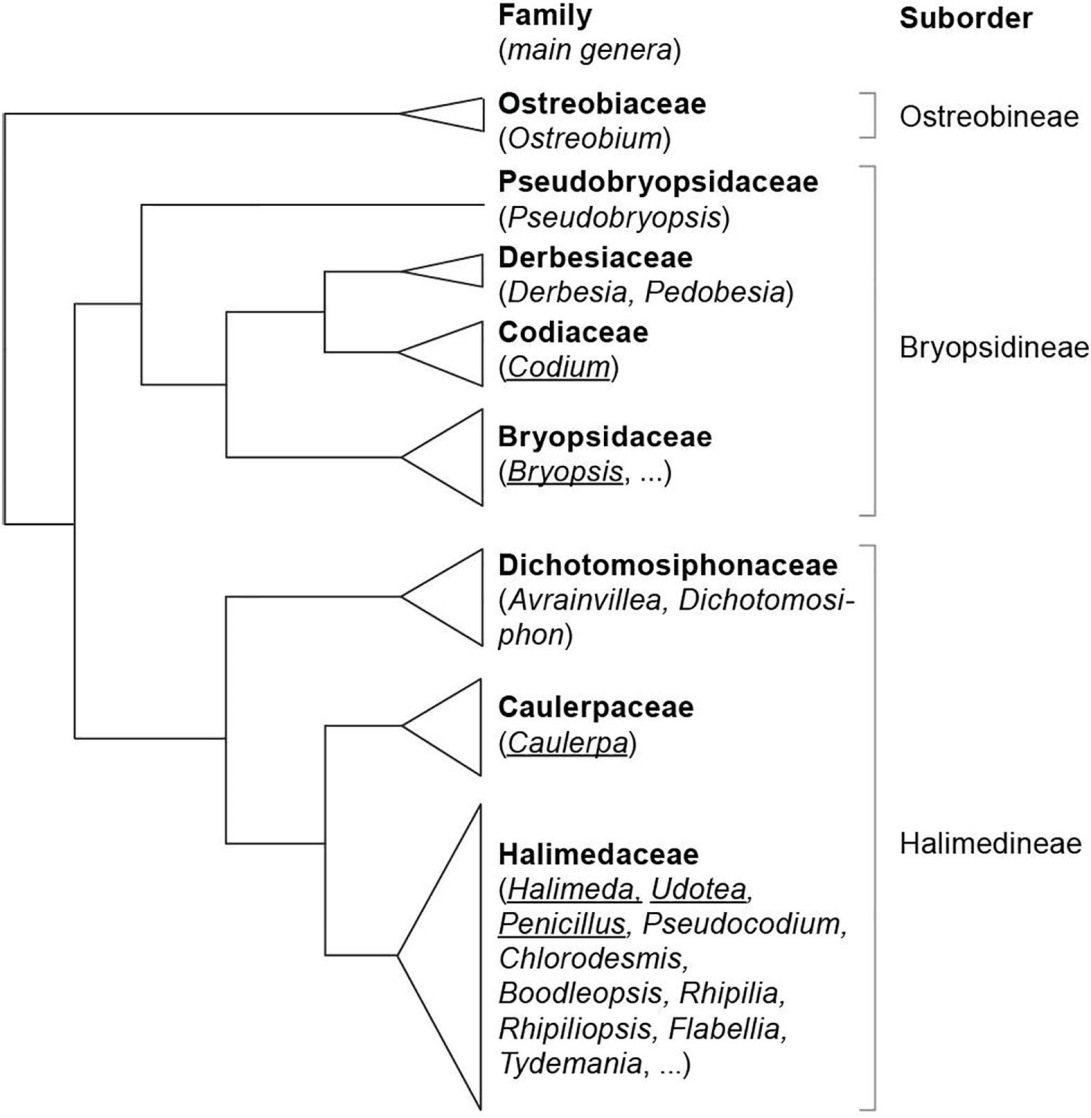
Phylogenetic relationships of the families and suborders of the Bryopsidales (based on Cremen et al., 2019). The genera for which data on sulfated polysaccharides is available, and which are discussed in this review, are underlined.

The Bryopsidales consist of three main clades, corresponding to the suborders Bryopsidineae, Halimedineae, and Ostreobineae (**Figure 3**), which are characterized based on differences in thallus morphology, reproduction and plastid types (Hillis-Colinvaux, 1984; Cremen et al., 2019).

The Bryopsidineae (including *Bryopsis*, *Codium*, and *Derbesia*) comprise the families Bryopsidaceae, Codiaceae, Derbesiaceae, and Pseudobryopsidaceae, with species inhabiting temperate to tropical marine waters. The Halimedineae (including *Caulerpa*, *Penicillus*, *Halimeda*, and *Udotea*), comprise the families Caulerpaceae, Halimedaceae, and Dichotomosiphonaceae, and are generally restricted to tropical and subtropical habitats. However, exceptions to these general distribution patterns occur, including the presence of some invasive *Caulerpa* species in temperate waters. Several species in this subclass (mainly in the Halimedaceae) are heavily calcified.

The structure of sulfated polysaccharides from the suborders Bryopsidineae and Halimedineae will be reviewed. No data on sulfated polysaccharides is currently available for the Ostreobineae.

### Bryopsidineae

The most widely studied genus with respect to its sulfated polysaccharides is *Codium*.

Love and Percival (1964) studied the water extracts from *Codium fragile*, which included 3-linked β-l-arabinopyranose and β-d-galactopyranose units. Sulfate was found on C-4 or C-6 of the galactose units, and on C-2 or C-3 of the arabinose residues. It was suggested that these sugars could be present in the same molecule, being arabinogalactans. More recently, extracts from *Codium* species were isolated and, eventually, fractionated and partially characterized because of their interest as anticoagulants (Ciancia et al., 2010). Most of these fractions contained galactose as major monosaccharide constituent, but a few contained arabinose as the major sugar, or had both.

**Figure 4:**
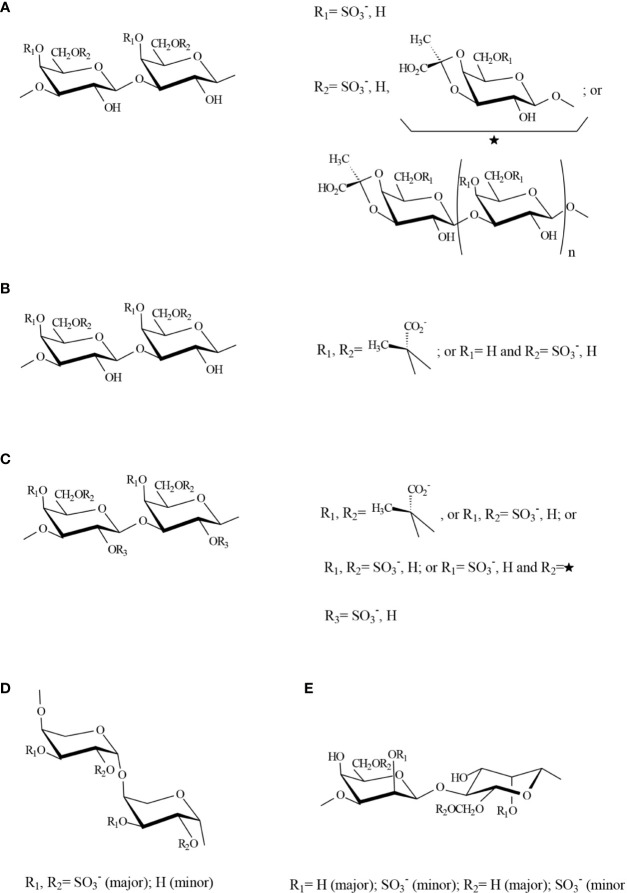
Possible structure for a fragment containing all the major structural features of sulfated polysaccharides **(A)** A galactan from *Codium* species, **(B)** An homogeneous fraction from *Bryopsis plumosa*, **(C)** Galactans from species in the Halimedaceae, **(D)** Pyranosic arabinan from *Codium* species, **(E)** Mannan from *Codium* species. Structures were proposed by the authors of this review based on the major components detected by Bilan et al., 2007; Estevez et al., 2009; Ciancia et al., 2012; Fernández et al., 2012; Fernández et al., 2013; Arata et al., 2015; Fernández et al., 2015.

A key work for understanding the actual structure of galactans from *Codium* was published by Bilan et al. (2007). In this paper, the structure of a sulfated, pyruvylated (1→3)(1→6)-β-d-galactan obtained from *C. yezoense* was exquisitely studied by chemical and spectroscopic methods. Acidic galactans of this kind were found in many other *Codium* species, including *C. fragile* (Ciancia et al., 2007; Estevez et al., 2009; Ohta et al., 2009; Li et al., 2016), *C. vermilara* (Ciancia et al., 2007; Fernández et al., 2013), *C. isthmocladum* (Farias et al., 2008; Araujo Sabry et al., 2019), *C. decorticatum* (Fernández et al., 2015), and *C. divaricatum* (Li et al., 2015). They showed highly ramified structures, which contained linear backbone segments of 3-linked β-d-galactopyranose residues connected by (1→6)-linkages, some of 3-linked residues were substituted at C-6, by short oligosaccharide chains also containing (1→3)- and (1→3,6)-linkages or by single β-d-galactopyranose residues. Sulfate groups were found mainly at C-4 and in minor amounts at C-6, confirming the study of Love and Percival (1964). The most unusual feature of these water-soluble galactans was the high pyruvate content, which was found to form mainly five-membered cyclic ketals with *O*-3 and *O*-4 of the non-reducing terminal galactose residues (*S*-isomer). However, a minor part of pyruvate formed six-membered cyclic ketals with *O*-4 and *O*-6 (*R*-configuration) (Bilan et al., 2006). These galactans are the only known algal polysaccharides containing five-membered cyclic pyruvate ketals studied until now. A possible structure for a fragment containing all the main structural features is shown in **Figure 4A**.

Although similar, galactans from different *Codium* species have important interspecific differences regarding the proportion of their structural units. For example, in samples of *C. fragile* from the Patagonian coast, 4,6-disulfated β-d-galactose units were found in significant quantities, and the presence of terminal 3,4-pyruvylated β-d-galactose 6-sulfate units was suggested (Estevez et al., 2009). The occurrence of the latter unit was proved by negative-ion electrospray tandem mass spectrometry of oligosaccharides obtained from the galactan from *C. divaricatum* in mild acid conditions (Li et al., 2016), and it was also detected recently in *C. isthmocladum* (Araujo Sabry et al., 2019). In the galactan from *C. decorticatum*, almost half of the pyruvate was forming a 6-membered ring with *O*-4 and *O*-6 of 3-linked β-d-galactose units (Fernández et al., 2015).

Sulfated arabinans with high anticoagulant activity were found in some *Codium* species, including *C. latum*, *C. dwarkense*, and others (Uehara et al., 1992; Siddhanta et al., 1999; Hayakawa et al., 2000), suggesting that these polymers, generally highly sulfated, are usual components of *Codium* cell walls. Later, Ciancia et al. (2007) showed that the major structural units in these polymers were 3-linked β-l-arabinopyranose highly sulfated on C-4, or C-2 and C-4 (**Figure 4D****)**. In particular, an almost persulfated linear arabinan was isolated from *C. vermilara* by precipitation in 0.1 M KCl (molar ratio arabinose:sulfate 1:1.8). This arabinan had a high anticoagulant activity by complex mechanisms, comprising direct and indirect inhibition of thrombin (Fernández et al., 2013). Arabinans from *C. decorticatum* were less sulfated and showed a high degree of branching at C-2, at least in part with single stubs of arabinose (Fernández et al., 2015). In the case of *C. fragile*, not only pyranosic 3-linked sulfated β-l-arabinans, but also furanosic non sulfated α-l-arabinan structures were detected, and they were associated to the presence of hydroxyproline rich glycoproteins (HRGP) (Estevez et al., 2009).

A third sulfated polysaccharide type was also found in water soluble extracts from *Codium* species and corresponded to 4-linked β-d-mannans, similar to those found as fibrillar components in these seaweeds, as well as plant mannans and galactomannans, but sulfated on C-2 and, in a lesser extent, on C-6 (Ciancia et al., 2007; Fernández et al., 2012; Fernández et al., 2015) (**Figure 4E****)**. A paper reporting immunomodulatory activity of a mannan from *C. fragile* postulated a structure corresponding to a 3-linked mannan comprising α- and β-units, sulfated on C-4 of the latter units (Tabarsa et al., 2013). However, the results, including NMR (nuclear magnetic resonance) spectroscopy and methylation analyses, suggest a structure similar to that found for the other *Codium* species.

As far as we know, the only other genus in this suborder for which sulfated polysaccharides have been studied in detail is *Bryopsis* (Ciancia et al., 2012). From a room temperature water extract, a linear 3-linked b-d-galactan, partially sulfated, mainly on C-6 and partially pyruvylated was isolated from *B. plumosa*. In this species, the pyruvylated units were part of the linear backbone, giving 4,6-*O*-(1´-(*R*)-carboxy)ethylidene-b-d-galactopyranose units (**Figure 4B**). The other polysaccharide obtained with water at room temperature was a furanosic, non-sulfated, mostly 2-linked β-l-arabinan, with ramifications on C-5. This structure is different to those detected in *C. fragile*, and further work should be done to confirm it. In addition, a rhamnan structure (17.4 mol% rhamnose) was detected as one of the major constituents in the hot water extract from the same seaweed. It comprised mainly 3-linked, possibly partially 4-sulfated, l-rhamnose units, but also 2-linked l-rhamnose residues. The fact that small amounts of uronic acids were present in these extracts, suggested that these rhamnans could be structurally related to sulfated polysaccharides from green seaweeds of the order Ulotrichales (Cassolato et al., 2008; Liu et al., 2018).

### Halimedineae

Several species in this suborder are calcified and give extremely low yields in sulfated polysaccharides, which may be the reason for the lack of information about their sulfated polysaccharides. As far as we know, the only structural study about sulfated polysaccharides in this algal group is that of Arata et al. (2015), who studied the water soluble extracts from three species, *Penicillus capitatus*, *Udotea flabellum* and *Halimeda opuntia* (Halimedaceae). They found that sulfated and pyruvylated β-d-galactans were the only soluble sulfated polysaccharides obtained in significant yields. Their backbones comprise 3-, 6-, and 3,6-linkages, constituted by major amounts of 3-linked 4,6-*O*-(1´-carboxy)ethylidene-d-galactopyranose units in part sulfated on C-2. Also possibly terminal 4,6-*O*-(1´-carboxy)ethylidene-d-galactopyranose residues were present in their structures, but in much lower amounts. Sulfation on C-2 was not found in galactans from other seaweeds of the Bryopsidales. In addition, a complex sulfation pattern, comprising 4-, 6-, and 4,6-disulfated galactose units was found, as in galactans from other species of this order (**Figure 4C**).

Mackie and Percival (1961) reported some structural features about the polysaccharides from three species of *Caulerpa*, *C. filiformis*, *C. racemosa*, and *C. sertularioides*. They all contained galactose as major monosaccharide, and significant quantities of mannose and xylose. Different batches of *C. filiformis* were investigated and in one of them, arabinose was also present. The authors attributed this difference to a seasonal variation, or to the fact that the latter sample was obtained in milder extraction conditions. They suggested that both galactose and mannose units were 3-linked, based on their resistance to periodate oxidation.

The sulfated heteropolysaccharide isolated from *Caulerpa taxifolia* comprised galactose, mannose, and xylose in molar ratio 16.4:5:1. 4-Linked xylose, 6-linked galactose, 4-linked mannose units, and non-reducing galactose end groups, which were all devoid of sulfate, were found. In addition, 4-linked galactose units sulfated at C-3 were also present. An oligosaccharide was isolated from the acid hydrolyzate, assigned to 4-*O*-(d-mannopyranosyl-4-*O*-d-gaIactopyranosyl)-d-mannopyranose (Prasada Rao and Venkata Rao, 1986). Later, a water soluble acidic heteroglycan sulfate, comprising arabinose, xylose, and galactose, was obtained from *Caulerpa racemosa* with a molecular mass of 80 kDa. It contained, *inter alia*, 3-linked galactose, terminal- and 4-linked xylose, and 4- and 3,4-linked arabinose residues. Sulfate groups, when present, were located at C-3 of 4-linked arabinose and C-6 of 3-linked galactose units (Chattopadhyay et al., 2007a).

Recently, a xylogalactomannan fraction containing galactose, mannose and xylose in molar ratio 2.4:2.2:1, and 21% sulfate was isolated from *C. lentillifera*. It consisted in 3-linked and terminal β-d-galactopyranose units, the former partially sulfated on C-6, 4-, 2,4-, and 2-linked β-d-mannopyranose units, the latter sulfated on C-3, and 4-linked 3-sulfated, and terminal β-d-xylopyranose units (Sun et al., 2018; Sun et al., 2020). This sample was reported to have a molecular weight of 3877.8 kDa, which is quite high for a sulfated polysaccharide, leading us to speculate that it could comprise several aggregated polysaccharides.

A water-soluble polysaccharide was obtained by hot-water extraction from *Caulerpa racemosa* var. *peltata* by Hao et al. (2019). After purification, it contained mannose as major monosaccharide component (92% of the total carbohydrates). The structural analysis reported a polysaccharide fraction comprising a backbone of 6-linked α-d-mannopyranose residues partially sulfated on C-3, with some 4-linked and 2-linked α-d-mannopyranose residues, and side chains comprising 4-linked β-d-galactopyranose residues.

Several papers appeared in the last few years, which reported biological activity of polysaccharides obtained from *Caulerpa* species, but these products were only characterized with respect to their molecular weight and monosaccharide composition, and no, or only little structural information was given (Chaves Filho et al., 2019; Albuquerque Ribeiro et al., 2020; Long et al., 2020; Zhang et al., 2020).

The lack of information about sulfated polysaccharides from such an important and widely distributed algal genus, comprising at least 75 species, some of which of them having invasive nature (Galil, 2007; Pierucci et al., 2019), is noteworthy. Also noteworthy is the variety of structures proposed in the few investigations published for the different species of the same genus. A revision of the structural features of cell wall polysaccharides from a variety of different macroalgae shows that a certain genus usually produces polysaccharides with only some variations from a general common structural pattern, which is characteristic for it. A lot of work is needed to clarify the structures of the whole system of sulfated polysaccharides biosynthesized by these organisms, as the small number of structural studies reported were usually carried out on a particular polysaccharide fraction, and there is not enough information about the other sulfated polysaccharides biosynthesized by the studied species. Likely, the whole polysaccharide system biosynthesized by *Caulerpa* species comprises variable quantities of sulfated β-d-galactans, pyranosic arabinans, and mannans. Xylose could be part of the galactan or arabinan structures, or another polymer.

### Dasycladales

Species in the Dasycladales have a siphonous architecture, with symmetrical thalli encrusted with calcium carbonate. Siphons contain either one macronucleus or millions of small nuclei. Species occur in marine tropical seas. Two families have traditionally been recognized based on differences in the reproductive structures, the Polyphysaceae (including *Acetabularia* and some other genera), and the paraphyletic Dasycladaceae (including genera like *Bornetella*, *Cymopolia*, and *Neomeris*) (Verbruggen et al., 2009).

Only a few reports are available about sulfated polysaccharides from the Dasycladales. Percival and Smestad (1972) studied the structural features of water soluble polysaccharides from stalks and caps of *Acetabularia crenulata*. They were able to determine the presence of 3-linked galactose 4-sulfate in major amounts, and also 6-sulfate, and 2-linked l-rhamnose, as the main structural features. Glucuronic acid, galactose, and rhamnose were present as end groups, indicating a highly branched molecule. Glucuronic acid was linked to both rhamnose and galactose. In addition, they found that galactose residues were linked together, and some galactoses were 4-*O*-methylated.

Cell walls from rhizoids, stalks, hairs, hair scars, apical septa, gametophores and gametangia from *A. acetabulum* were prepared, but the polysaccharides were not separated according to their water solubility, but analyzed directly by NMR spectroscopy and monosaccharide composition (Dunn et al., 2007). In these conditions, the major monosaccharide component was in all cases mannose, arising in major amounts from the fibrillar cell wall polysaccharide components. Glucose, deriving from cellulose, was also detected, mainly in gametophores and gametangia (haploid structures). Additionally, galactose, rhamnose, xylose, and arabinose were found in different but always small amounts. Although the authors performed methylation analysis, the predominance of mannose in these extracts precludes further conclusions.

The very limited information about these polymers makes it impossible to draw conclusions, however, we speculate that these seaweeds could biosynthesize both sulfated 3-linked β-d-galactans or arabinogalactans and glucuronorhamnans. This speculation is based on the results of methylation analysis of samples from different parts of the thallus from *Acetabularia acetabulum*, that show, in addition to important amounts of mannans and cellulose, other monosaccharides, as rhamnose and glucuronic acid (Dunn et al., 2007) and the detail paper by Percival and Smestad (1972), which suggests the presence of 3-linked 4-sulfated galactan structures, similar to those of *Codium*, in *Acetabularia crenulata*. These polysaccharides could be distributed in the cell walls of each seaweed structure in different quantities, according to their requirements. Moreover, it was found that the amount of rhamnose in various wall regions roughly correlated with the degree of flexibility of that anatomical region. The proportion of rhamnose was the greatest in hairs, the most flexible region, often undulating in the moving seawater (Dunn et al., 2007). These authors associated the monosaccharide composition determined with cell wall polymers from flowering plants, but they can also be associated to cell wall polysaccharide components from phylogenetically closer organisms, as other green algae.

### Cladophorales

**Figure 5:**
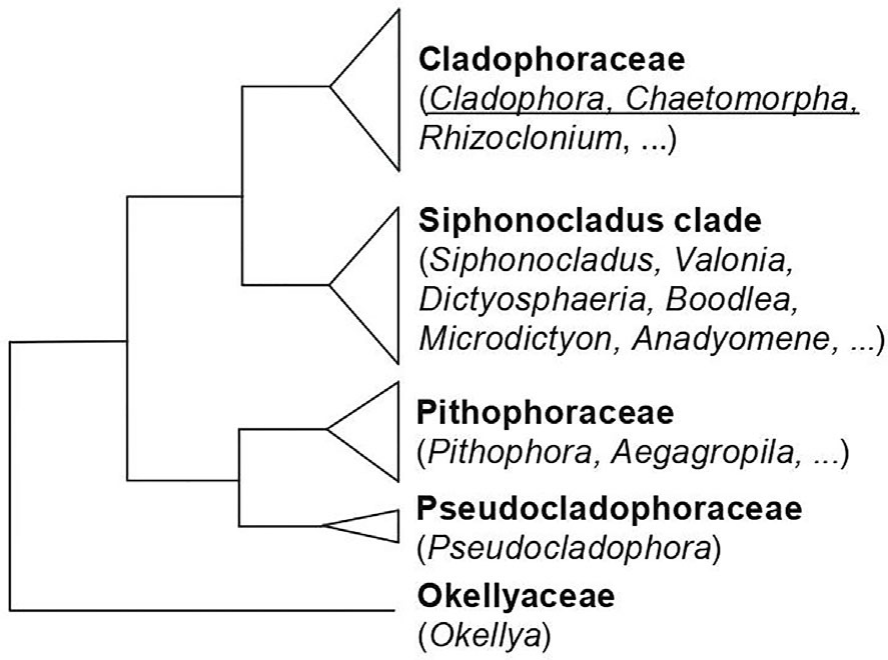
Phylogenetic relationships of the families of Cladophorales, based on Leliaert et al., 2009 and Boedeker et al., 2012. The genera for which data on sulfated polysaccharides is available, and which are discussed in this review are underlined.

Species of Cladophorales occur in a wide variety of marine, brackish and freshwater habitats from tropical to polar environments. Thallus organization ranges from simple, branched or unbranched filaments to more complex architectural types. The order comprises five main family-level clades (**Figure 5**). The two largest clades are the Cladophoraceae, including genera with relative simple thallus morphology such as *Cladophora*, *Chaetomorpha* and *Rhizoclonium*, and occurring in marine to freshwater habitats, and the *Siphonocladus* clade, including a wider range of thallus architectures, and occurring in marine habitats. Cell wall polysaccharides have, as far as we know, only been investigated in species of the Cladophoraceae.

The extensive studies of Percival and coworkers about cell wall polysaccharides from green seaweeds include the determination of fine structural details of sulfated polysaccharides from *Cladophora rupestris*, which comprised arabinose, galactose, xylose, rhamnose, and glucose in molar ratio of 3.7:2.8:1.0:0.4:0.2, as well as 8% protein and 19.6% sulfate (as SO_3_Na) (Fischer and Percival, 1957). A highly branched structure, with xylose and galactose units at the non-reducing ends of the side chains and galactose, arabinose, and rhamnose residues occurring in the inner part of the molecules was proposed. The evidence of 6-linked and/or 6-sulfated galactofuranose units obtained in this study is noteworthy, as furanosic galactose containing polysaccharides were only found in some green microalgae species associated with lichens, as *Trebouxia* sp. and *Myrmecia biatorellae* (Trebouxiales, Trebouxiophyceae). These polysaccharides have a main backbone of β-d-galactofuranose units, with different side chains, constituted by single stubs of β-d-galactofuranose and/or more complex branched structures, which comprise, in some cases, α-l-rhamnopyranose units (Cordeiro et al., 2005; Cordeiro et al., 2012; Cordeiro et al., 2013).

Partial hydrolysis experiments of the sulfated polysaccharides from *C. rupestris* led to the separation and characterization of the following fragments: l-arabinose 3-sulfate, d-galactose 6-sulfate, 3-linked and 6-linked d-galactobioses, 4- or 5-linked l-arabinobiose 3-sulfate, 4-linked d-xylobiose, a mixture of trisaccharides containing sulfated galactose and arabinose, and a mixture of pentasaccharides in which the molar ratio of arabinose to galactose was 4:1 (Hirst et al., 1965; Johnson and Perival, 1969; Bourne et al., 1970). In addition, preliminary studies were also done on the sulfated polysaccharides from *Chaetomorpha capillaris* and *Ch. linum*, which gave a predominance of arabinose as monosaccharide component (Hirst et al., 1965). For these seaweeds, no further studies were carried out at that time due to the difficulties to get enough material. Later, it was found that arabinose was also the predominant monosaccharide component of the sulfated polysaccharides from *Ch. anteninna* (58% of the purified polysaccharide), and it was in the pyranosic form, giving 4-linked arabinopyranose partially sulfated on C-2, while 3- and 4-linked galactopyranose units were also present in important amounts (39%) and small quantities of 4-linked rhamnose were also detected (Venkata Rao and Sri Ramana, 1991).

The same authors studied the sulfated polysaccharides from *Cladophora socialis* obtained by extraction with dilute acid (Sri Ramana and Venkata Rao, 1991). A molar ratio galactose:arabinose:xylose of 4.5:3.0:1.0 and 16.9% of sulfate were determined. In this case, the 4-linked arabinose units were found to be 3-sulfated, but no galactose in the furanose form was detected, we think it could have been lost due to its lability in acidic extraction conditions. In addition, galactose units were found to be 3-linked and sulfated on C-4 or C-4 and C-6.

**Figure 6:**
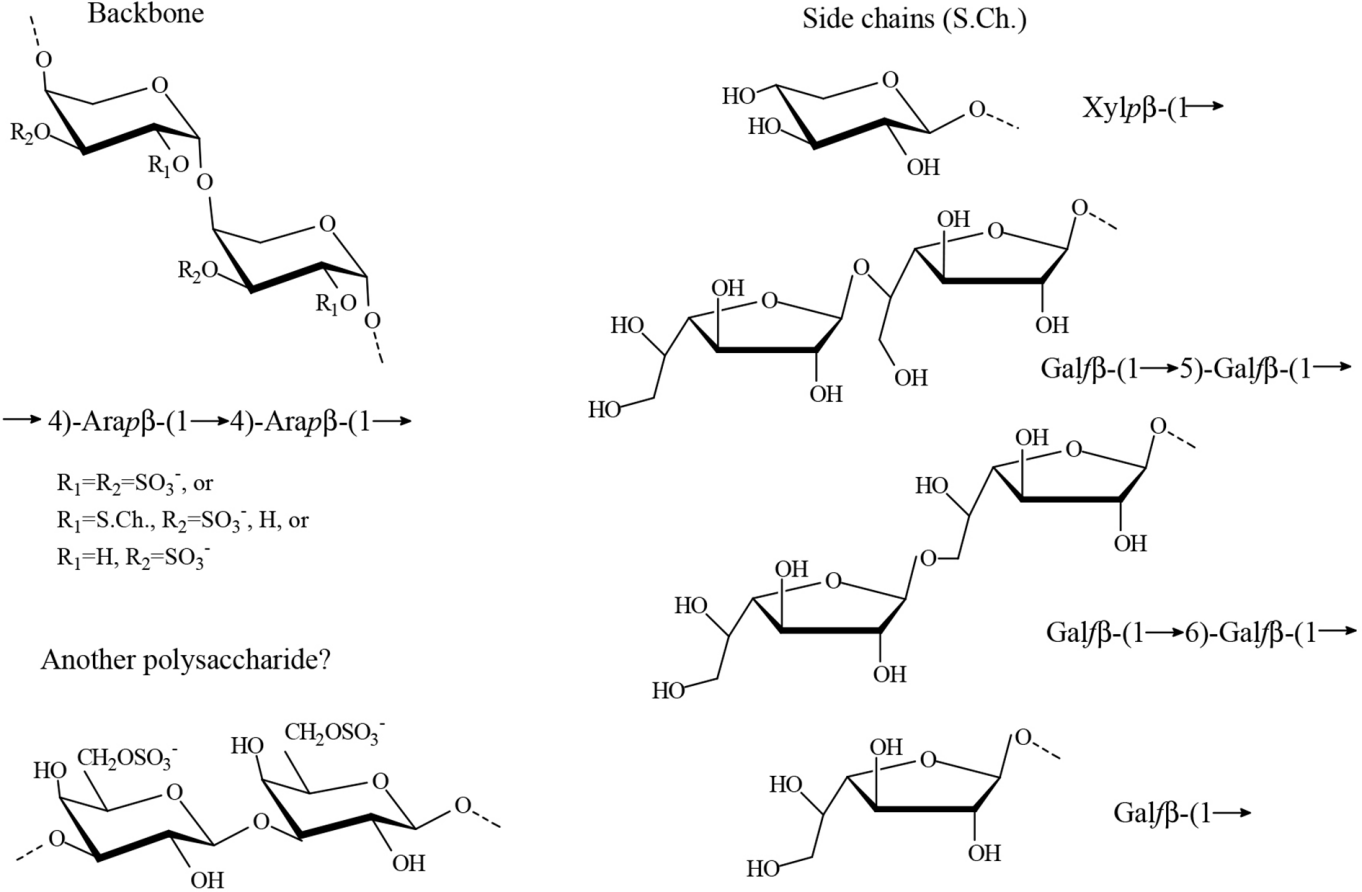
Proposed major structures identified in the water extracts from *Cladophora falklandica* (Arata et al., 2016). Furanosic galactan side chains could have different number of units, and each chain could have mixed linkages. In some fractions, 5,6-linked Gal*f* units were detected. It is speculated that the pyranosic galactan structure constitutes an independent polysaccharide, but this remains to be confirmed.

The studies carried out by Arata et al. (2016) on the water soluble sulfated polysaccharides from the seaweed *Cladophora falklandica* were also in agreement with the classical work (Percival and McDowell, 1981), but the authors were able to determine some structural features not reported before using modern spectroscopic methods in addition to the chemical determinations. It was found that the major sulfated polysaccharides from this species were xylogalactoarabinans constituted by a backbone of 4-linked β-l-arabinopyranose units partially sulfated mainly on C-3 and also on C-2. Besides, partial glycosylation mostly on C-2 with single stubs of β-d-xylopyranose, or single stubs of β-d-galactofuranose or short chains comprising (1→5)- and/or (1→6)-linkages, was also found (**Figure 6**). In addition 3-linked β-d-galactopyranose units sulfated on C-6 were also detected in minor amounts in some of the fractions. It was not possible to establish whether they were part of the major polysaccharides or if they constituted another polysaccharides present in minor amounts. Small amounts of rhamnose were also present in all these fractions.

Similar polysaccharide structures were found for the freshwater *Cladophora surera*, although only traces of galactose in the furanosic form were detected. The presence of sulfated polysaccharides in a freshwater green macroalga of the Cladophorales could be, in this case, an adaptation to transient desiccation and changes in ionic strength of its environment (Arata et al., 2017a).

In addition, Surayot et al. (2016) proposed for the water soluble polysaccharides from *Cladophora glomerata*, another freshwater alga phylogenetically very close to *C. surera* (Arata et al., 2017a), a backbone of 4-linked α-l-arabinopyranose units, with galactose and xylose residues as branches and sulfate groups linked to C-2 of the arabinose units. They also reported terminal arabinofuranose units. It is important to note, that the configuration of C-1-C-4 of α-l-arabinopyranose is the same as that of β-d-galactopyranose, and the configuration of C-1-C-4 of β-l-arabinopyranose is the same as that of α-d-galactopyranose (**Supplementary Figure S1**), so the anomeric signals reported by these authors should correspond to the β-anomer of l-arabinopyranose. It is thus reasonable to believe that their experimental results are in agreement with structures proposed by Percival and coworkers (Hirst et al., 1965; Johnson and Perival, 1969; Bourne et al., 1970) and Arata et al. (2016; 2017a).

While writing this review, a paper about the structure of a sulfated polysaccharide fraction from *Chaetomorpha linum* was published, with a new structure constituted by 6-linked β-D-galactopyranose and 5-linked α-L-arabinofuranose residues with sulfate groups on C-2 or C-3 of the latter units and on C-2 or C-4 of 6-linked β-D-galactopyranose residues (Qin et al., 2020). This structure was determined by chemical and spectroscopic analyses. As only one fraction was studied, it is not known if the remaining carbohydrate material has similar structures to those published before for other phylogenetically close algal species.

A pyranosic 4-linked β-l-arabinan sulfated on C-3 was also isolated from the green seaweed *Ulva* (*Enteromorpha*) *clathrata* (Ulvales) in a pure form, and only minor amounts of rhamnose and glucuronic acid were found (Qi et al., 2012). This arabinan has the same backbone and major sulfation pattern as that of *Cladophora* species, as well as that reported for *Caulerpa racemosa* (Chattopadhyay et al., 2007a, see above). This result is unexpected, considering that it is well documented that green algae from the Ulvales biosynthesize uronate-rich sulfated polysaccharides, known as ulvans, composed of rhamnose, xylose, glucuronic acid, and iduronic acid (Lahaye and Robic, 2007; Cosenza et al., 2017), but not arabinose. However, there is no doubt regarding the isolated structure, determined by the chemical and spectroscopic analyses presented in this paper. Moreover, as indicated by the authors, this high arabinose-containing sulfated polysaccharide has different chemical characteristics from the sulfated polysaccharides isolated from species of the Ulvales, which should belong to the uronic acid rich polysaccharide group, as defined by Percival (1979). In the last years, many reports confirmed that seaweeds of this order, including *Ulva lactuca*, *U. intestinalis*, *U. prolifera*, *U. linza*, and *U. compressa*, biosynthesize major amounts of sulfated glucuronorhamnans (Chattopadhyay et al., 2007b; Lahaye and Robic, 2007; Wang et al., 2013; Yu et al., 2017; Li et al., 2018). In these reports, arabinose was not detected, or if so, only in trace amounts. If the algal species determination was correct, a revision of the classification of sulfated polysaccharides from green seaweeds regarding their phylogenetic positions could be evaluated. In addition, it should be considered that only the major fraction obtained from the hot water extract of *U. clathrata* by anion exchange chromatography was analyzed, and no information was given regarding the other polysaccharides of the extract (Qi et al., 2012).

Another possible exception was reported for the structure of sulfated polysaccharides from *Spongomorpha indica* (Ulotrichales). Their backbone was composed by 4-linked galactose units, terminal, 3-linked and 2-linked arabinofuranose units, as well as 4-linked arabinopyranose units were detected, and xylose was present as branches. Sulfate groups were found on some of the arabinose units at C-2 and on some of the galactose units at C-2 and C-3 (Venkata Rao et al., 1991).

## Concluding Remarks

By reviewing the diversity of sulfated polysaccharides from cell walls of coenocytic ulvophytes in the orders Bryopsidales, Dasycladales, and Cladophorales in view of their phylogenetic relationships, we identified important knowledge gaps related to the diversity of sulfated polysaccharides in green seaweeds. The knowledge on the structures of cell wall polysaccharides, especially the sulfated polysaccharides, biosynthesized by them is very limited, even for many common and widely distributed species, and, in very few cases, the whole system of cell wall sulfated polysaccharides was investigated. In addition, the species that have been investigated are distributed unevenly among the orders, families and genera of the Ulvophyceae. Several families within the orders Bryopsidales, Dasycladales, and Cladophorales have not been studied, and the same is true for several smaller orders of Ulvophyceae, including the Trentepohliales, Ignatiales, Scotinosphaerales, Chlorocystidales, and Oltmannsiellopsidales (**Figure 2**). This is because most of the studies were not carried out with a phylogenetic perspective, but with the aims of finding biologically active compounds that could have interest as potential pharmacological drugs, or with other commercial interests. Information on these understudied groups, however, will be invaluable to reconstruct the evolution of sulfated polysaccharides in the Ulvophyceae, and could potentially result in the detection of novel bioactive compounds. This is plausible given the antiquity of the ulvophycean lineages, which likely diverged in the Proterozoic (Del Cortona et al., 2020), providing plenty of time to evolve novel bioactive compounds. In addition ulvophycean species occur in a wide diversity of habitats, ranging from fully marine environments to freshwater and strictly terrestrial habitats (Škaloud et al., 2018), which likely has an important effect on polysaccharide structure.

Until now, no useful rheological properties were found for these sulfated polysaccharides, as those of galactans from red seaweeds (carrageenans and agarose) that make these compounds industrially produced as hydrocolloids (Gomez et al., 2020). Moreover, their small yields make this possible application unlikely. Conversely, many studies have sought to find different biological activities of these compounds. For example, anticoagulant activity of polysaccharides from *Codium* species was thoroughly investigated for many years (Ciancia et al., 2010; Fernández et al., 2014; Li et al., 2015; Araujo Sabry et al., 2019). More recently, anticoagulant activity of sulfated polysaccharides from other coenocytic green algae was also explored, including detailed studies about their mechanisms of action (Arata et al., 2015; Arata et al., 2016; Arata et al., 2017b; Qin et al., 2020). In addition, some other interesting biological activities were found, including proinflamatory activity (Tabarsa et al., 2013), anti-inflamatory activity (Sun et al., 2020), macrophage activation (Surayot et al., 2016), antioxidant activity (Hamzaoui et al., 2020), immunostimulatory activity (Lee et al., 2010; Sun et al., 2018; Hao et al., 2019), and antiviral activity (Pujol et al., 2012). Thus, it seems that the potential application of these polysaccharides could arise from their biological action.

Although a lot of work is still needed, the available information shows important characteristics that define the different coenocytic green algal groups, up to order and also the family level. **Table 1** gives a picture of the possible generalizations that can be made at this stage.

**Table 1:** General characteristics of cell wall polysaccharides from coenocytic green algae.

Order		Bryopsidales		Dasycladales	Cladophorales
**Cytomorphological type**		Siphonous		Siphonous	Siphonocladous
**Habitat**		Marine (except for one genus)		Marine	Marine and Freshwater
**Major fibrillar component**		(1→4)-β-mannans or (1→3)-β- xylans and small amounts of cellulose		(1→4)-β-mannans	Cellulose (Chitin)?
**Uronic acids**		Absent (or present in very low quantities)		Present	Absent
**Sulfated ** **polysaccharides**	**Backbone**	**Suborder Bryopsidineae**	**Suborder Halimedineae**	***Acetabularia***	**Cladophoraceae**
	**Galactan**	***Codium***: **-3,6-linked-β-galactan** sulfated on C-4 and C-6, **3,4** (also **4,6-) pyruvylated** ***Bryopsis***: -linear **3-linked-β-galactan** sulfated on C-6), **4,6-pyruvylated**	**Halimedaceae:** **-3,6-linked-β-galactan 4,6-pyuvylated** Galp **sulfated on C-2** (major), Galp sulfated on C-4 and/or C-6 **Caulerpaceae:** **-3-linked-β-galactan** sulfated on C-6	**-3-linked-β-galactan** (sulfated on C-4 and C-6)	**-3-linked-β-galactan** (sulfated on C-6) **-6-linked β-galactan** sulfated on C-2 or C-4
	**Arabinan**	***Codium***: **-pyranosic 3-linked-β-arabinan** sulfated on C-2 and C-4, or on C-4 -Ara*f* from HRGPs ***Bryopsis***: -2,5-linked-Ara*f*	**Caulerpaceae:** **-pyranosic 4-linked-arabinan** sulfated on C-3	-Sulfated arabinogalactan	**-Pyranosic 4 linked-β-arabinan** (sulfated on C-3 or C-2 and C-3), with **Gal*f***, Rha and Xyl -5-linked α-Ara*f* sulfated on C-2 or C-3
	**Other**	***Codium:*** **-4-linked-β-mannan** sulfated on C-2 and/or C-6 **Bryopsis**: **-Glucuronorhamnan**	**Caulerpaceae:** **- 6-linked β-mannan (galactomannan)?** **-Xylose on Ara*p* or Gal*p*?** **- 2- and/or 4-linked β-mannan sulfated on C-3** **- α-mannan?**	**Glucuronorhamnan**	**Small amounts of Rha -Glucuronorhamnan?**

Cell walls are very complex and diverse supramolecular structures. Their macromolecular components and arrangement have been elucidated for some groups of organisms as the flowering plants (Gibeaut and Carpita, 1993; Park and Cosgrove, 2012). Conversely, algae comprise of many distantly related groups of organisms, for some of which cell wall structures are better understood than others (Popper et al., 2011; Domozych et al., 2012). Particularly, cell wall architecture from green macroalgae of the Ulvophyceae has been investigated in only a few cases (Lahaye and Robic, 2007; Estevez et al., 2009; Fernández et al., 2010; Ciancia et al., 2012; Fernández et al., 2014; Fernández et al., 2015).

It is evident from **Table 1** that some important structural features of sulfated polysaccharides are shared between the Cladophorales and Bryopsidales, in spite of their phylogenetic distance, as the presence of 3-linked β-d-galactans and 3- or 4-linked β-l-arabinans that are not usually found in those from the Ulvales and Ulotrichales.

Pyranosic 3-linked β-d-galactans are found in many different organisms, as cell wall arabinogalactan proteins (AGP) and pectins from higher plants, but pyranosic β-l-arabinans are not. Moreover, l-arabinose is a constituent of many different plant cell wall components, but, although the pyranose form of l-arabinose is thermodynamically more stable, in these polymers, it occurs mostly in the furanose form (Fernández et al., 2013). More precisely, L-arabinopyranose units were found as part of side chains in Type II arabinogalactans from pectins and in some xyloglucans from flowering plants (Caffal and Mohnen, 2009; Tuomivaara et al., 2015), usually as terminal non reducing units, but not as oligo- or poly-saccharide sequences. So, it is tempting to speculate that pyranosic arabinans are a specific character of cell walls from ancestor organisms of the Ulvophyceae that are still present in some lineages, but were lost during evolution in some others, and it could be related to the evolution of green seaweeds and land plants.

There are other structural features that are characteristic of a certain order, as the presence of pyruvic acid substituting some of the β-d-galactose units in β-d-galactans from the Bryopsidales, or the presence of β-d-galactofuranose in side chains of arabinans from the Cladophorales.

In the case of the Bryopsidales, there is enough information to look further into the suborder and family level of structural relationships between sulfated galactans. The presence of the pyruvic acid substituent of β-d-galactans forming a five-membered cyclic ketal with *O*-3 and *O*-4 of non-reducing terminal galactose residues was only found in the Codiaceae, while pyruvic acid ketals forming a six-membered ring with *O*-4 and *O*-6 of some of the 3-linked β-D-galactose units of the galactan backbone, were found in all the species of Bryopsidales in which this structural unit was searched for (Fernández et al., 2014).

In this review, the major sulfated polysaccharides have been considered. In most, if not in all, cases, small amounts of other monosaccharide constituents suggest that there could be some other polysaccharides, minor constituents of the cell wall, the nature of which remains elusive. For example, when the sulfated polysaccharide systems from *Cladophora falklandica* and *C. surera* were studied, minor amounts of rhamnose were detected (Arata et al., 2016; Arata et al., 2017a). It is not known if it is derived from a rhamnan present in minor amounts, or if it is a minor constituent of the major water soluble polysaccharides obtained from these algae. In addition, a sulfated rhamnan structure was detected in the hot water extract from *Bryopsis plumosa* in considerable amounts (Ciancia et al., 2012). These observations can only be made when all the sulfated polysaccharides from a certain species are investigated, and could make us speculate that ulvophyte green algae have the enzymes to biosynthesize all of these polysaccharides, but only produce some through biosynthetic regulating factors yet to be unveiled. This speculation could explain the presence of polysaccharides others than those expected in some cases, as in *Ulva clathrata* and *Spongomorpha indica* (Venkata Rao et al., 1991; Qi et al., 2012).

Another possibility in view of the latest findings regarding evolution of ulvophyte green algae (Del Cortona et al., 2020) is that not only morphological characters, such as siphonous thallus architecture, but also some structural features of the cell wall, including some sulfated polysaccharides structures, could have appeared independently during evolution of these green algae as a result of similar environmental selective pressures, or more likely as a response to increasingly enlarged cells in the course of evolution of the Bryopsidales and Dasycladales.

## Author Contributions

MC and PF analyzed and discussed the polysaccharides structures. FL analyzed and dicussed the phylogenetic relationships. MC programmed the review structure. MC and FL integrated the whole analysis and wrote the manuscript.

## Funding

This work was supported by the National Research Council of Argentina (CONICET) (PIP 11220130100762CO 2014-2016, PU-E 2016 22920160100068CO) and the University of Buenos Aires (UBACYT 2018-2021, 20020170100347BA).

## Conflict of Interest

The authors declare that the research was conducted in the absence of any commercial or financial relationships that could be construed as a potential conflict of interest.

## References

[B1] Albuquerque Ribeiro N. Vasconcelos Chaves H. Conceição Rivanor R. L. Rochado Val D. Limade Assis L. Dantas Silveira F. (2020). Sulfated polysaccharide from the green marine algae *Caulerpa racemosa* reduces experimental pain in the rat temporomandibular joint. Int. J. Biol. Macromol. 150, 253–260. 10.1016/j.ijbiomac.2020.01.272 32004610

[B2] Aquino R. S. Landeira-Fernandez A. M. Valente A. P. Andrade L. R. Mourão P. A. (2005). Occurrence of sulfated galactans in marine angiosperms: evolutionary implications. Glycobiology 15, 11–20. 10.1093/glycob/cwh138 15317737

[B3] Arata P. X. Quintana I. Canelón D. J. Vera B. E. Compagnone R. S. Ciancia M. (2015). Chemical structure and anticoagulant activity of highly pyruvylated sulfated galactans from tropical green seaweeds of the order Bryopsidales. Carbohydr. Polym. 122, 376–386. 10.1016/j.carbpol.2014.10.030 25817682

[B4] Arata P. X. Quintana I. Raffo M. P. Ciancia M. (2016). Novel sulfated xylogalactoarabinans from green seaweed *Cladophora falklandica*: chemical structure and action on the fibrin network. Carbohydr. Polym. 154, 139–150. 10.1016/j.carbpol.2016.07.088 27577905

[B5] Arata P. X. Alberghina J. Confalonieri V. Errea M. I. Estevez J. M. Ciancia M. (2017a). Sulfated polysaccharides in the green macroalga *Cladophora surera* not linked to salinity adaptation. Front. Plant Sci. 8, 1927. 10.3389/fpls.2017.01927 29181012 PMC5694217

[B6] Arata P. X. Genoud V. Lauricella A. M. Ciancia M. Quintana I. (2017b). Alterations of fibrin networks mediated by sulfated polysaccharides from green seaweeds. Thromb. Res. 159, 1–4. 10.1016/j.thromres.2017.09.014 28934617

[B7] Araujo Sabry D. Lima Cordeiro S. Ferreira Silva C. H. Cunha Farias E. H. Lanzi Sassaki G. Bonciani Nader H. (2019). Pharmacological prospection and structural characterization of two purified sulfated and pyruvylated homogalactans from green algae *Codium isthmocladum*. Carbohydr. Polym 222, 115010. 10.1016/j.carbpol.2019.115010 31320102

[B8] Bilan M. II Vinogradova E. V. Shashkov A. S. Usov A. I. (2006). Isolation and preliminary characterization of a highly pyruvylated galactan from *Codium yezoense* (Bryopsidales, Chlorophyta). Bot. Mar. 49, 259–262. 10.1515/BOT.2006.029

[B9] Bilan M. II Vinogradova E. V. Shashkov A. S. Usov A. II (2007). Structure of a highly pyruvylated galactan sulfate from the pacific green alga *Codium yezoense* (Bryopsidales, Chlorophyta). Carbohydr. Res. 342, 586–596. 10.1016/j.carres.2006.11.008 17134684

[B10] Boedeker C. O’Kelly C. J. Star W. Leliaert F. (2012). Molecular phylogeny and taxonomy of the *Aegagropila* clade (Cladophorales, Ulvophyceae), including the description of *Aegagropilopsis* gen. nov. and *Pseudocladophora* gen. nov. J. Phycol. 48, 808–825. 10.1111/j.1529-8817.2012.01145.x 27011097

[B11] Bourne E. J. Johnson P. G. Perival E. (1970). The water-soluble polysaccharides of *Cladophora rupestris*.Part IV. Autohydrolysis, methylation of the partly desulphated material and correlation with the results of Smith degradation. J. Chem. Soc C. 1561–1569. 10.1039/J39700001561

[B12] Caffal K. H. Mohnen D. (2009). The structure, function, and biosynthesis of plant cell wall pectic polysaccharides. Carbohydr. Res. 344, 1879–1900. 10.1016/j.carres.2009.05.021 19616198

[B13] Cassolato J. E. F. Noseda M. D. Pujol C. A. Pellizzari F. M. Damonte E. B. Duarte M. E. R. (2008). Chemical structure and antiviral activity of the sulphated heterorhamnan isolated from the green seaweed *Gayralia oxysperma*. Carbohydr. Res. 343, 3085–3095. 10.1016/j.carres.2008.09.014 18845298

[B14] Chattopadhyay K. Adhikari U. Lerouge P. Ray B. (2007a). Polysaccharides from *Caulerpa racemosa*: Purification and structural features. Carbohydr. Polym. 68, 407–415. 10.1016/j.carbpol.2006.12.010

[B15] Chattopadhyay K. Mandal P. Lerouge P. Driouich A. Ghosal P. Ray B. (2007b). Sulphated polysaccharides from Indian samples of *Enteromorpha compressa* (Ulvales, Chlorophyta): Isolation and structural features. Food Chem. 104, 928–935. 10.1016/j.foodchem.2006.12.048

[B16] Chaves Filho G. P. de Sousa A. F. G. Viana R. L. S. Rocha H. A. O. Batistuzzo de Medeiros S. R. Moreira S. M. G. (2019). Osteogenic activity of non-genotoxic sulfated polysaccharides from the green seaweed *Caulerpa sertularioides*. Algal. Res. 14, 101546. 10.1016/j.algal.2019.101546

[B17] Ciancia M. Quintana I. Vizcargüenaga M. I. Kasulin L. de Dios A. Estevez J. M. (2007). Polysaccharides from the green seaweeds *Codium fragile* and *C. vermilara* with controversial effects on hemostasis. Int. J. Biol. Macromol. 41, 641–649. 10.1016/j.ijbiomac.2007.08.007 17920674

[B18] Ciancia M. Quintana I. Cerezo A. S. (2010). Overview of anticoagulant activity of sulfated polysaccharides from seaweeds in relation to their structures, focusing on those of green seaweeds. Curr. Med. Chem. 17, 2503–2529. 10.2174/092986710791556069 20491645

[B19] Ciancia M. Alberghina J. Arata P. X. Benavides H. Leliaert F. Verbruggen H. (2012). Characterization of cell wall polysaccharides of the coencocytic green seaweed *Bryopsis plumosa* (Bryopsidaceae, Chlorophyta) from the Argentine coast. J. Phycol. 48, 326–335. 10.1111/j.1529-8817.2012.01131.x 27009722

[B20] Cordeiro L. M. C. Carbonero E. R. Sassaki G. L. Reis R. A. Stocker-Wörgötter E. Gorin P. A. J. (2005). A fungus-type β-galactofuranan in the cultivated *Trebouxia* photobiont of the lichen *Ramalina gracilis*. FEMS Microbiol. Lett. 244, 193–198. 10.1016/j.femsle.2005.01.040 15727840

[B21] Cordeiro L. M. C. Beilke F. Bettim F. L. Reinhardt V. F. Rattmann Y. D. Iacomini M. (2012). (1→2) and (1→6)-linked β-D-galactofuranan of microalga *Myrmecia biatorellae*, symbiotic partner of *Lobaria linita*. Carbohydr. Polym. 90, 1779– 1785. 10.1016/j.carbpol.2012.07.069 22944447

[B22] Cordeiro L. M. C. Beilke F. Reinhardt V. F. Sassaki G. L. Iacomini M. (2013). Rhamnogalactofuranan from the microalga *Myrmecia biatorellae*, symbiotic partner of *Lobaria linita*. Phytochemistry 94, 254–259. 10.1016/j.phytochem.2013.06.008 23850078

[B23] Cosenza V. A. Navarro D. A. Ponce N. M. A. Stortz C. A. (2017). “Seaweed Polysaccharides: Structure and Applications,” in Industrial Applications of Renewable Biomass Products Past, Present and Future. Eds. D´Accorso N. B. Goyanes S. N. (Cham, Switzerland: Springer Nature), 75–116. 10.1007/978-3-319-61288-1

[B24] Cremen M. C. M. Leliaert F. West J. Lam D. W. Shimada S. Lopez-Bautista J. M. (2019). Reassessment of the classification of Bryopsidales (Chlorophyta) based on chloroplast phylogenomic analyses. Mol. Phylogenet. Evol. 130, 397–405. 10.1016/j.ympev.2018.09.009 30227214

[B25] Del Cortona A. Leliaert F. (2018). Molecular evolution and morphological diversification of ulvophytes (Chlorophyta). Perspect. Phycol. 5, 27–43. 10.1127/pip/2017/0075

[B26] Del Cortona A. Jackson C. J. Bucchini F. Van Bel M. D’hondt S. Škaloud P. (2020). Neoproterozoic origin and multiple transitions to macroscopic growth in green seaweeds. PNAS 117, 2551–2559. 10.1073/pnas.1910060117 31911467 PMC7007542

[B27] Deniaud-Bouët E. Hardouin K. Potin P. Kloareg B. Hervé C. (2017). A review about brown algal cell walls and fucose-containing sulfated polysaccharides: Cell wall context, biomedical properties and key research challenges. Carbohydr. Polym. 175, 395–408. 10.1016/j.carbpol.2017.07.082 28917882

[B28] Domozych D. S. Ciancia M. Fangel J. U. Dalgaard Mikkelsen M. Ulvskov P. Willats W. G. T. (2012). The cell walls of green algae: a journey through evolution and diversity. Front. Plant Sci. 3, 82. 10.3389/fpls.2012.00082 22639667 PMC3355577

[B29] Dunn E. K. Shoue D. A. Huang X. Kline R. E. MacKay A. L. Carpita N. C. (2007). Spectroscopic and biochemical analysis of regions of the cell wall of the unicellular “mannan weed”, *Acetabularia acetabulum*. Plant Cell Physiol. 48, 122–133. 10.1093/pcp/pcl053 17169920

[B30] Estevez J. M. Ciancia M. Cerezo A. S. (2004). The system of galactans of the red seaweed, *Kappaphycus alvarezii*, with emphasis on its minor constituents. Carbohydr. Res. 339, 2575–2592. 10.1016/j.carres.2004.08.010 15476719

[B31] Estevez J. M. Fernández P. V. Kasulin L. Dupree P. Ciancia M. (2009). Chemical and *in situ* characterization of macromolecular components of the cell wallsfrom the green seaweed *Codium fragile*. Glycobiol 19, 212–228. 10.1093/glycob/cwn101 18832454

[B32] Falshaw R. Bixler H. J. Johndro K. (2001). Structure and performance of commercial kappa-2 carrageenans extracts I. Structure analysis. Food Hydrocoll. 15, 441–452. 10.1016/S0268-005X(01)00066-2

[B33] Farias E. H. C. Pomin V. H. Valente A. P. Nader H. B. Rocha H. A. O. Mourão P. A. S. (2008). A preponderantly 4-sulfated, 3-linked galactan from the green alga *Codium isthmocladum*. Glycobiol 18, 250–259. 10.1093/glycob/cwm139 18174311

[B34] Fernández P. V. Ciancia M. Miravalles A. B. Estevez J. M. (2010). Cell-wall polymer mapping in the coenocytic macroalga *Codium vermilara* (Bryopsidales, Chlorophyta). J. Phycol. 46, 456–465. 10.1111/j.1529-8817.2010.00821.x 27020016

[B35] Fernández P. V. Estevez J. M. Cerezo A. S. Ciancia M. (2012). Sulfated β-d-mannan from the green seaweed *Codium vermilara*. Carbohydr. Polym 87, 916–919. 10.1016/j.carbpol.2011.06.063 34663054

[B36] Fernández P. V. Quintana I. Cerezo A. S. Caramelo J. J. Pol-Fachin L. Verli H. (2013). Anticoagulant activity of a unique sulphated pyranosic (1→3)-β-l-arabinan through direct interaction with thrombin. J. Biol. Chem. 288, 223–233. 10.1074/jbc.M112.386441 23161548 PMC3537017

[B37] Fernández P. V. Arata P. X. Ciancia M. (2014). “Polysaccharides from Codium species: Chemical structure and biological activity. Their role as components of the cell wall in sea plants,” in Advances in Botanical Research (Vol. 71). Ed. Bourgougnon N. (Oxford, Great Britain: Elsevier), 253–278. 10.1016/B978-0-12-408062-1.00009-3

[B38] Fernández P. V. Raffo M. P. Alberghina J. Ciancia M. (2015). Polysaccharides from the green seaweed *Codium decorticatum*. Structure and cell wall distribution. Carbohydr. Polym. 117, 836–844. 10.1016/j.carbpol.2014.10.039 25498707

[B39] Fischer I. S. Percival E. (1957). The water-soluble polysaccharides of *Cladophora rupestris*. J. Chem. Soc. 2666–2675. 10.1039/jr9570002666

[B40] Galil B. (2007). Loss or gain? Invasive aliens and biodiversity in the Mediterranean Sea. Mar. Pollut. Bull. 55, 314–322. 10.1016/j.marpolbul.2006.11.008 17222869

[B41] Gibeaut D. M. Carpita N. C. (1993). Structural models of primary cell wall in flowering plants: consistency of molecular structure with the physical properties of the walls during growth. Plant J. 3, 1–30. 10.1111/j.1365-313x.1993.tb00007.x 8401598

[B42] Gomez L. P. Alvarez C. Zhao M. Tiwari U. Curtin J. Garcia-Vaquero M. (2020). Innovative processing strategies and technologies to obtain hydrocolloids from macroalgae for food applications. Carbohydr. Polym. 248, 116784. 10.1016/j.carbpol.2020.116784 32919572

[B43] Hamzaoui A. Ghariani M. Sellem I. Hamdi M. Feki A. Jaballi I. (2020). Extraction, characterization and biological properties of polysaccharide derived from green seaweed *Chaetomorpha linum* and its potential application in Tunisian beef sausages. Int. J. Biol. Macromol. 148, 1156–1168. 10.1016/j.ijbiomac.2020.01.009 31917214

[B44] Hao H. Han Y. Yang L. Hu L. Duan X. Yang X. (2019). Structural characterization and immunostimulatory activity of a novel polysaccharide from green alga *Caulerpa racemosa* var *peltata*. Int. J. Biol. Macromol. 134, 891–900. 10.1016/j.ijbiomac.2019.05.084 31100398

[B45] Hayakawa Y. Hayashi T. Lee J.-B. Srisomporn P. Maeda M. Ozawa T. (2000). Inhibition of thrombin by sulphated polysaccharides isolated from green seaweeds. Biochim. Biophys. Acta 1543, 86–94. 10.1016/S0167-4838(00)00193-X 11087944

[B46] Hillis-Colinvaux L. (1984). “Systematics of the Siphonales,” in Systematics of the Green Algae. Eds. Irvine D. E. G. John D. M. (London, Great Britain: Academic Press), 271–291.

[B47] Hirst E. Mackie W. Percival E. (1965). The water-soluble polysaccharides of *Cladophora rupestris* and *Chaetomorpha* spp.Part II ). J. Chem. Soc. 2958–2967. 10.1039/jr9650002958

[B48] Huizing H. J. Rietema H. (1975). Xylan and mannan as cell wall constituents of different stages in the life-histories of some siphoneous green algae. Br. Phycol. J. 10, 13–16. 10.1080/00071617500650021

[B49] Huizing H. J. Rietema H. Sietsma J. H. (1979). Cell wall constituents of several siphoneous green algae in relation to morphology and taxonomy. Br. Phycol. J. 14, 25–32. 10.1080/00071617900650051

[B50] Johnson P. G. Perival E. (1969). Water-soluble polysaccharides of *Cladophora rupestris*. Part III. Smith degradation. J. Chem. Soc. C. 906–909. 10.1039/J39690000906

[B51] Kidgell J. T. Magnusson M. de Nysa R. Glasson C. R. K. (2019). Ulvan: A systematic review of extraction, composition and function. Algal. Res. 39, 101422. 10.1016/j.algal.2019.101422

[B52] Kloareg B. Quatrano R. S. (1988). Structure of the cell wall of marine algae and ecophysiological functions of the matrix polysaccharides. Oceanogr. Mar. Biot. Annu. Rev. 26, 259–315.

[B53] Lahaye M. Robic A. (2007). Structure and functional properties of ulvan, a polysaccharide from green seaweed. Biomacromolecules 8, 1765–1774. 10.1021/bm061185q 17458931

[B54] Lee J. B. Ohta Y. Hashashi K. Hashashi T. (2010). Immunostimulating effects of a sulfated galactan from Codium fragile. Carbohydr. Polym. 345, 1452–1454. 10.1016/j.carres.2010.02.026 20362278

[B55] Leliaert F. Rueness J. Boedeker C. Maggs C. A. Cocquyt E. Verbruggen H. (2009). Systematics of the marine microfilamentous green algae *Uronema curvatum* and *Urospora microscopica* (Chlorophyta). Europ. J. Phycol. 44, 487–496. 10.1080/09670260903229540

[B56] Leliaert F. Smith D. R. Moreau H. Herron M. D. Verbruggen H. Delwiche C. F. (2012). Phylogeny and Molecular Evolution of the Green Algae. Crit. Rev. Plant Sci. 31, 1–46. 10.1080/07352689.2011.615705 2012.

[B57] Li N. Mao W. Yan M. Liu X. Xia Z. Wang S. (2015). Structural characterization and anticoagulant activity of a sulfated polysaccharide from the green alga *Codium divaricatum*. Carbohydr. Polym. 121, 175–182. 10.1016/j.carbpol.2014.12.036 25659687

[B58] Li N. Mao W. Liu X. Wang S. Xia Z. Cao S. (2016). Sequence analysis of the pyruvylated galactan sulfate-derived oligosaccharides by negative-ion electrospray tandem mass spectrometry. Carbohydr. Res. 433, 80–88. 10.1016/j.carres.2016.07.018 27471831

[B59] Li X. Xiong F. Liu Y. Liu F. Hao Z. Chen H. (2018). Total fractionation and characterization of the water-soluble polysaccharides isolated from *Enteromorpha intestinalis*. Int. J. Biol. Macromol. 111, 319–325. 10.1016/j.ijbiomac.2018.01.018 29325743

[B60] Liu X. Cao S. Qin L. He M. Sun H. Yang Y. (2018). A sulfated heterorhamnan with novel structure isolated from the green alga *Monostroma angicava*. Carbohydr. Res. 466, 1–10. 10.1016/j.carres.2018.06.010 29986167

[B61] Long H. Gu X. Zhou N. Zhu Z. Wang C. Liu X. (2020). Physicochemical characterization and bile acid-binding capacity of water-extract polysaccharides fractionated by stepwise ethanol precipitation from *Caulerpa lentillifera*. Int. J. Biol. Macromol. 150, 654–661. 10.1016/j.ijbiomac.2020.02.121 32061693

[B62] Love J. Percival E. (1964). The polysaccharides of the green seaweed *Codium fragile*: Part II. The water-soluble sulphated polysaccharides. J. Chem. Soc. 3338–3345. 10.1039/JR9640003338

[B63] Mackie I. M. Percival E. (1961). Polysaccharides from the green seaweeds of *Caulerpa* spp. Part III. Detailed study of the water-soluble polysaccharides of *C. filiformis*: comparison with the polysaccharides synthesised by C. racemosa, and XXXC. sertularioides. J. Chem. Soc, 3010–3015. 10.1039/JR9610003010

[B64] Maeda M. Kuroda K. Iriki Y. Chihara M. Nisizawa K. Miwa T. (1966). Chemical nature of major cell wall constituents of *Vaucheria* and *Dichotomosiphon* with special reference to their phylogenetic positions. Bot. Mag. (Tokio) 79, 634–643. 10.15281/jplantres1887.79.634

[B65] Mine I. Menzel D. Okud K. (2008). Morphogenesis in giant-celled algae. Int. Rev. Cell Mol. Biol. 266, 37–83. 10.1016/S1937-6448(07)66002-X 18544492

[B66] Ohta Y. Lee J.-B. Hayashi K. Hayashi T. (2009). Isolation of sulfated galactan from *Codium fragile* and its antiviral effect. Biol. Pharm. Bull. 32, 892–898. 10.1248/bpb.32.892 19420760

[B67] Pangestuti R. Kurnianto D. (2017). “Green Seaweeds-Derived Polysaccharides Ulvan: Occurrence, Medicinal Value and Potential Applications,” in Seaweed Polysaccharides. Isolation, Biological and Biomedical Applications. Eds. Venkatesan J. Anil S. Kim S. K. (Oxford, Great Britain: Elsevier), 205–221. 10.1016/B978-0-12-809816-5.00011-6

[B68] Park Y. B. Cosgrove D. J. (2012). A Revised Architecture of Primary Cell Walls Based on Biomechanical Changes Induced by Substrate-Specific Endoglucanases. Plant Physiol. 158, 1933–1943. 10.1104/pp.111.192880 22362871 PMC3320196

[B69] Pearlmutter N. L. Lembi C. A. (1980). Structure and composition of *Pithophora oedogonia* (Chlorophyta) cell walls. J. Phycol. 16, 602–616. 10.1111/j.1529-8817.1980.tb03079.x

[B70] Percival E. McDowell R. H. (1981). “Algal walls: composition and biosynthesis,” in Encyclopedia of Plant Physiology, vol. 13B Eds. Tanner W. Loewus F. A. (Berlin, Germany: Springer), 277–316.

[B71] Percival E. Smestad B. (1972). Carbohydrates of Acetabularia species: Part II. A. crenulata acid polysaccharide. Carbohydr. Res. 25, 299–312. 10.1016/S0008-6215(00)81640-7

[B72] Percival E. (1979). The polysaccharides of green, red and brown seaweeds: Their basic structure, biosynthesis and function. Br. Phycol. J. 14, 103–117. 10.1080/00071617900650121

[B73] Pérez Recalde M. Canelón D. J. Compagnone R. S. Matulewicz M. C. Cerezo A. S. Ciancia M. (2016). Carrageenan and agaran structures from the red seaweed *Gymnogongrus tenuis*. Carbohydr. Polym. 136, 1370–1378. 10.1016/j.carbpol.2015.10.007 26572482

[B74] Pierucci A. De La Fuente G. Cannas R. Chiantore M. (2019). A new record of the invasive seaweed *Caulerpa cylindracea* Sonder in the South Adriatic Sea. Heliyon 5, e02449. 10.1016/j.heliyon.2019.e02449 31687554 PMC6819781

[B75] Pomin V. H. Mourão P. A. (2008). Structure, biology, evolution, and medical importance of sulfated fucans and galactans. Glycobiology 18, 1016–1027. 10.1093/glycob/cwn085 18796647

[B76] Popper Z. A. Michel G. Hervé C. Domozych D. S. Willats W. G. Tuohy M. G. (2011). Evolution and diversity of plant cell walls: from algae to flowering plants. Ann. Rev. Plant Biol. 62, 567–590. 10.1146/annurev-arplant-042110-103809 21351878

[B77] Prasada Rao N.V.S.A.V. Venkata Rao E. (1986). Structural features of the sulphated polysaccharide from a green seaweed, *Caulerpa taxifolia*. Phytochemistry 25, 1645–1647. 10.1016/S0031-9422(00)81227-3

[B78] Pujol C. A. Ray S. Ray B. Damonte E. B. (2012). Antiviral activity against dengue virus of diverse classes of algal sulfated polysaccharides. Int. J. Biol. Macromol. 51, 412–416. 10.1016/j.ijbiomac.2012.05.028 22652218

[B79] Qi X. Mao W. Gao Y. Chen Y. Chen Y. Zhao C. (2012). Chemical characteristic of an anticoagulant-active sulfated polysaccharide from *Enteromorpha clathrata*. Carbohydr. Polym. 90, 1804–1810. 10.1016/j.carbpol.2012.07.077 22944450

[B80] Qin L. He M. Yang Y. Fu Z. Tang C. Shao Z. (2020). Anticoagulant-active sulfated arabinogalactan from *Chaetomorpha linum*: Structural characterization and action on coagulation factors. Carbohydr. Polym. 242, 116394. 10.1016/j.carbpol.2020.116394 32564857

[B81] Siddhanta A. K. Shanmugam M. Mody K. H. Goswami A. M. Ramabat B. K. (1999). Sulphated polysaccharides of *Codium dwarkense* Boergs. from the westcoast of India: Chemical composition and blood anticoagulant activity. Int. J. Biol. Macromol. 26, 151–154. PII S0141-8130(99)00079-3. 10.1016/S0141-8130(99)00079-3 10517522

[B82] Škaloud P. Rindi F. Boedeker C. Leliaert F. (2018). Freshwater Flora of Central Europe, Vol 13: Chlorophyta: Ulvophyceae (Süßwasserflora von Mitteleuropa, Bd. 13: Chlorophyta: Ulvophyceae) (Berlin, Heidelberg: Springer Spektrum), 288 pp. 10.1007/978-3-662-55495-1

[B83] Sri Ramana K. Venkata Rao E. (1991). Structural features of the sulphated polysaccharide from a green seaweed, *Cladophora socialis*. Phytochemistry 30, 259–262. 10.1016/0031-9422(91)84133-D 1369326

[B84] Stiger-Pouvreau V. Bourgougnon N. Deslandes E. (2016). Carbohydrates from seaweeds”, Seaweed in Health and Disease Prevention. Eds. Fleurence J. Levine I. (Cambridge, Massachusetts: Academic Press, Elsevier), 223–274. 10.1016/B978-0-12-802772-1.00008-7

[B85] Sun Y. Gong G. Guo Y. Wang Z. Song S. Zhu B. (2018). Purification, structural features and immunostimulatory activity of novel polysaccharides from *Caulerpa lentillifera*. Int. J. Biol. Macromol. 108, 314–323. 10.1016/j.ijbiomac.2017.12.016 29222013

[B86] Sun Y. Liu Z. Song S. Zhu B. Zhao L. Jiang J. (2020). Anti-inflammatory activity and structural identification of a sulfated polysaccharide CLGP4 from *Caulerpa lentillifera*. Int. J. Biol. Macromol. 146, 931–938. 10.1016/j.ijbiomac.2019.09.216 31730965

[B87] Surayot U. Lee J. H. Kanongnuch C. Peerapornpisal Y. Park W. J. You S. G. (2016). Structural characterization of sulfated arabinans extracted from *Cladophora glomerata* Kützing and their macrophage activation. Biosci. Biotechnol. Biochem. 80, 972–982. 10.1080/09168451.2015.1132149 26818722

[B88] Tabarsa M. Karnjanapratum S. Cho M. Kim J.-K. You S. G. (2013). Molecular characteristics and biological activities of anionic macromolecules from *Codium fragile*. Int. J. Biol. Macromol. 59, 1–12. 10.1016/j.ijbiomac.2013.04.022 23597705

[B89] Tuomivaara S. T. Yaoi K. O’Neill M. A. York W. S. (2015). Generation and structural validation of a library of diverse xyloglucan-derived oligosaccharides, including an update on xyloglucan nomenclature. Carbohydr. Res. 402, 56–66. 10.1016/j.carres.2014.06.031 25497333

[B90] Udayan A. Arumgam M. Pandey A. (2017). “Nutraceuticals From Algae and Cyanobacteria,” in Algal Green Chemistry. Recent Progress in Biotechnology. Eds. Rastogi R. P. Madamwar D. Pandey A. (Oxford, Great Britain: Elsevier B.V), 65–89.

[B91] Uehara T. Takeshita M. Maeda M. (1992). Studies on anticoagulant-active arabinan sulfates from the green alga, *Codium latum*. Carbohydr. Res. 2, 309–311. 10.1016/0008-6215(92)80100-F 1473111

[B92] Usov A. I. (2011). Polysaccharides of the red algae. Adv. Carbohydr. Chem. Biochem. 65, 115–217. 10.1016/B978-0-12-385520-6.00004-2 21763512

[B93] Vavilala S. L. D´Souza J. S. (2015). “Algal Polysaccharides and their biological applications,” in Marine Algae Extracts: Processes, Products, and Applications. Eds. Kim S. K. Chojnacka K. (Hoboken, New Jersey: John Wiley & Sons), 411–451. 10.1002/9783527679577.ch26

[B94] Venkata Rao E. Sri Ramana K. (1991). Structural studies of a polysaccharide isolated from the green seaweed *Chaetomorpha anteninna*. Carbohydr. Res. 217, 163–170. 10.1016/0008-6215(91)84126-Y 1797398

[B95] Venkata Rao P. Prasada Rao N.V.S.A.V. Sri Ramana K. (1991). Structural features of the sulphated polysaccharide from a green seaweed, *Spongomorpha indica*. Phytochemistry 30, 1183–1186. 10.1016/s0031-9422(00)95199-9 1367384

[B96] Verbruggen H. Ashworth M. LoDuca S. T. Vlaeminck C. Cocquyt E. Sauvage T. (2009). A multi-locus time-calibrated phylogeny of the siphonous green algae. Mol. Phylogenet. Evol. 50, 642–653. 10.1016/j.ympev.2008.12.018 19141323

[B97] Wang X. Zhang Z. Yao Z. Zhao M. Qi H. (2013). Sulfation, anticoagulant and antioxidant activities of polysaccharide from green algae *Enteromorpha linza*. Int. J. Biol. Macromol. 58, 225–230. 10.1016/j.carbpol.2012.07.077 23587999

[B98] Wutz M. Zetsche K. (1976). Zur Biochimie und Regulation des Heteromorphen Generationswechsels der Grünalge. Planta 129, 211–216. 10.1007/BF00398259 24430958

[B99] Yu Y. Li Y. Du C. Mou H. Wang P. (2017). Compositional and structural characteristics of sulfated polysaccharide from *Enteromorpha prolifera*. Carbohydr. Polym. 165, 221–228. 10.1016/j.carbpol.2017.02.011 28363544

[B100] Zhang M. Zhao M. Qing Y. Luo Y. Xia G. Li Y. (2020). Study on immunostimulatory activity and extraction process optimization of polysaccharides from *Caulerpa lentillifera*. Int. J. Biol. Macromol. 143, 677–684. 10.1016/j.ijbiomac.2019.10.042 31730975

[B101] Zulkifly S. B. Graham J. M. Young E. B. Mayer R. J. Piotrowski M. J. Smith I. (2013). The genus *Cladophora* Kütsing (Ulvophyceae) as globally distributed ecological engineer. J. Phycol. 49, 1–17. 10.1111/jpy.12025 27008383

