# Supplementary material for: Diversity of Sulfated Polysaccharides From Cell Walls of Coenocytic Green Algae and Their Structural Relationships in View of Green Algal Evolution
Source: Front Plant Sci. 2020 Sep 29;11:554585. doi: 10.3389/fpls.2020.554585 (PMC7550628; doi:10.3389/fpls.2020.554585)
Supplement: Supplementary file 1 [file DataSheet_1.pdf]

Supplementary Figure 1. The configuration of C-1-C-4 of β-L-arabinopyranose is the same as that of α-D-galactopyranose. (1) In the α-anomer, the hydroxyl group is at the same side that the one that gives the name to the series (C-5 in this case). (2) The groups placed in the right side in the Fischer projection, are placed down in the Haworth projection, and vice versa. (3) The same as (1), but in this case the hydroxyl group that gives the name to the series is the one linked to C-4. It is clear from (2) and (4) that the anomeric signals in polysaccharides having both monosaccharides will be similar.

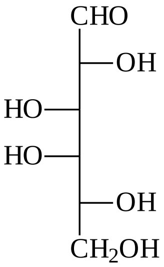

D-Gal
Fischer projection

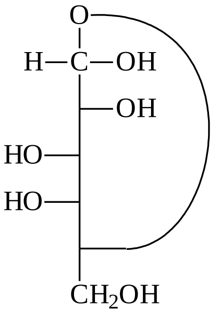

α-D-Gal*p*
Fischer projection
(1)

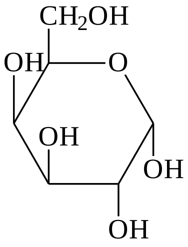

α-D-Gal*p*
Haworth projection
(2)

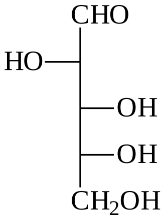

D-Ara
Fischer projection

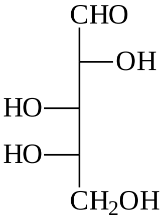

L-Ara
Fischer projection

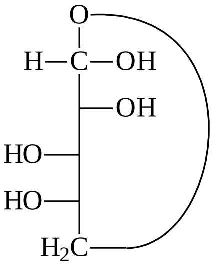

β-L-Ara*p*
Fischer projection
(3)

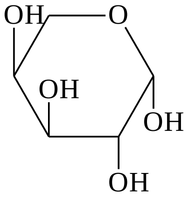

β-L-Ara*p*
Haworth projection
(4)
